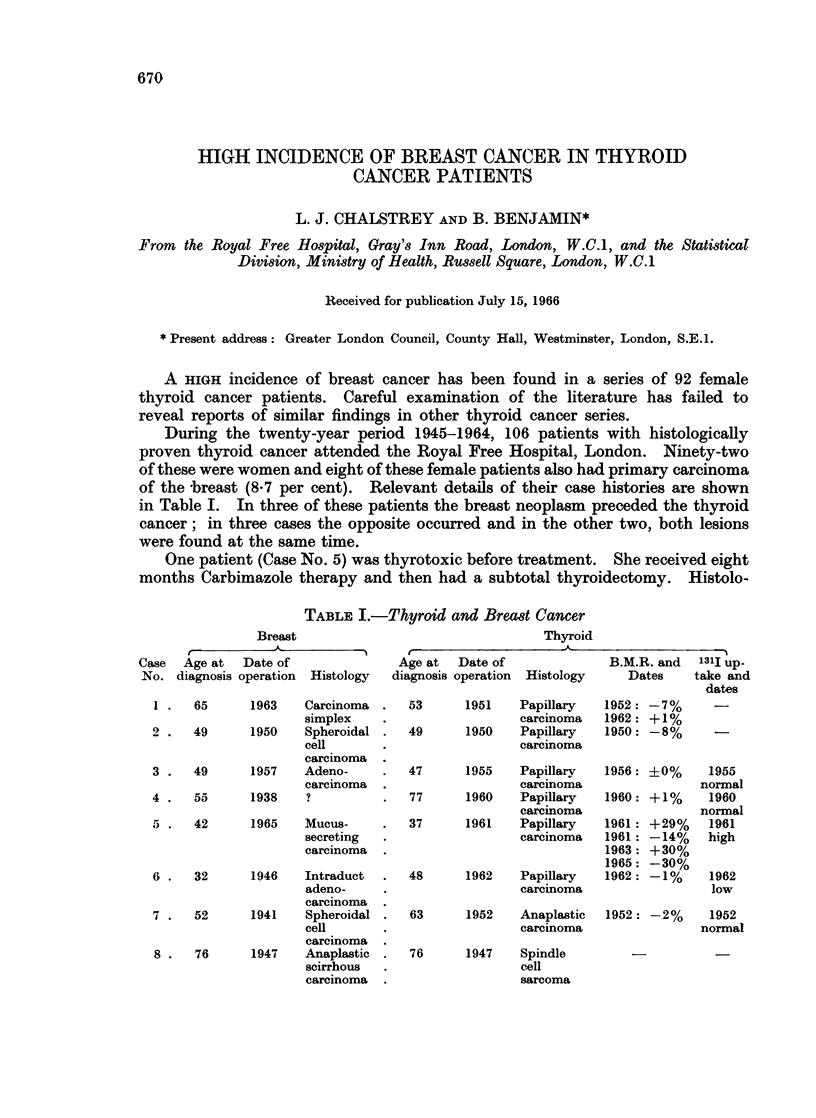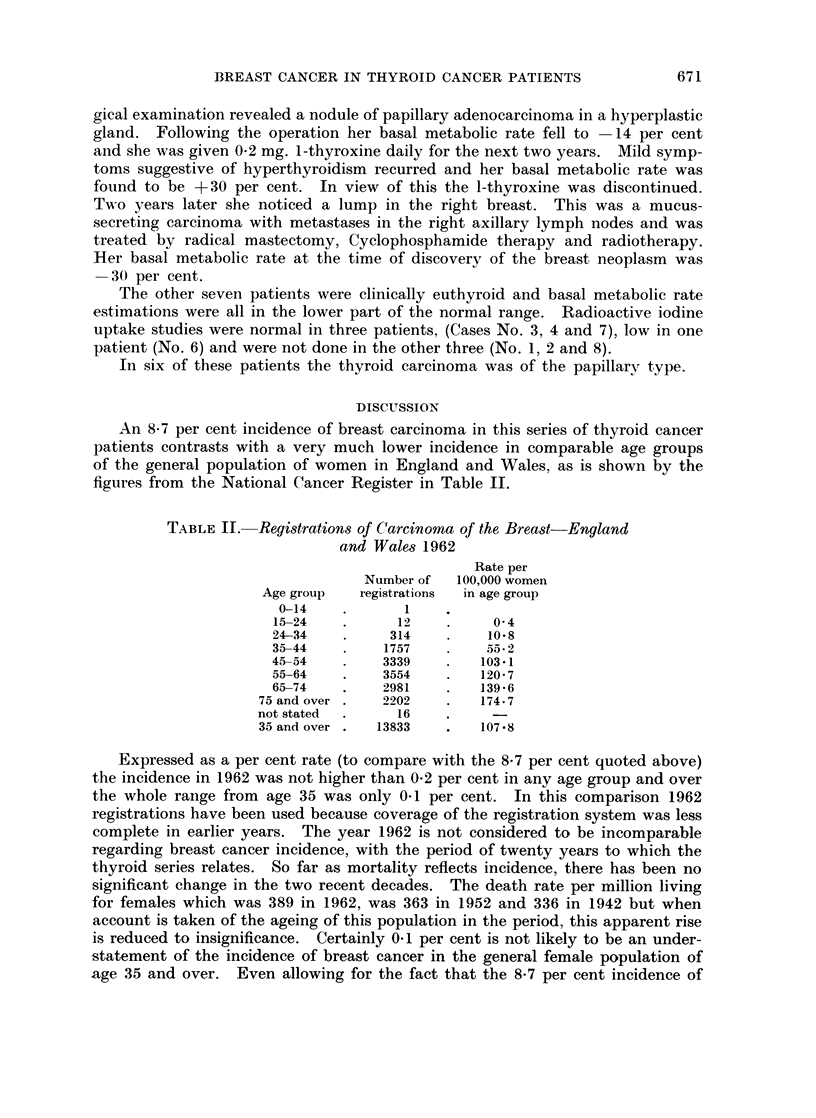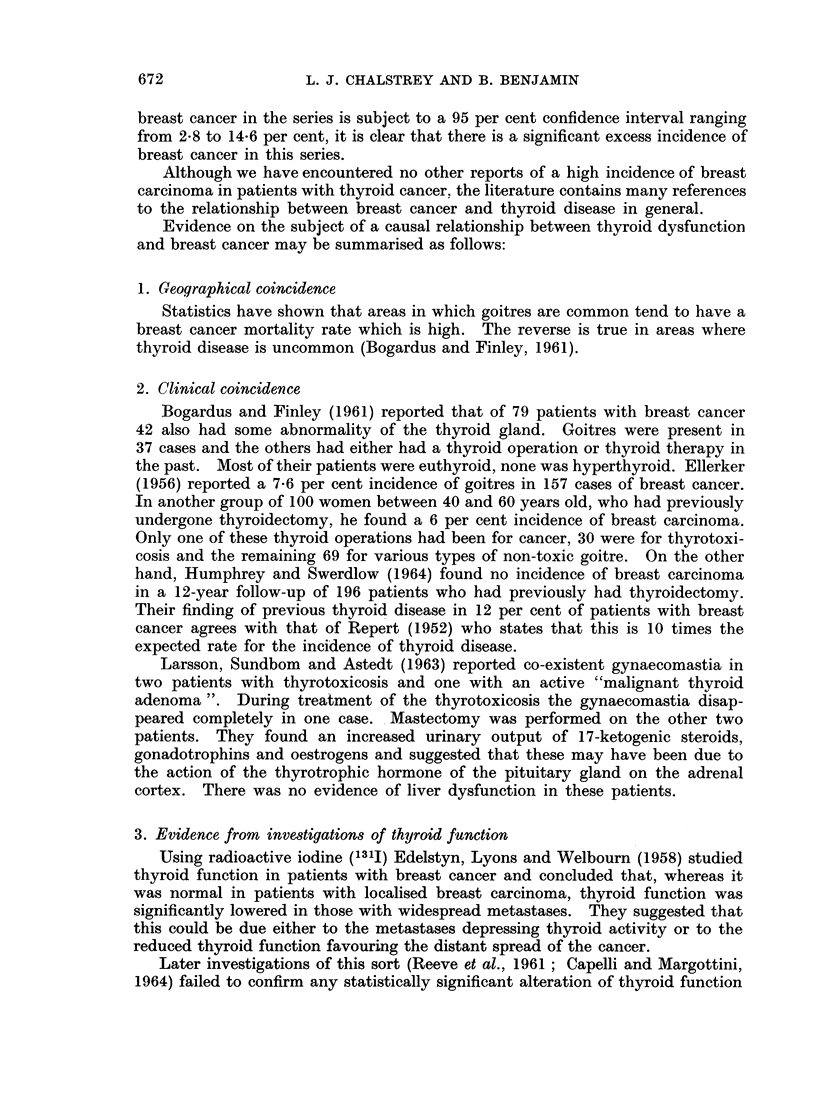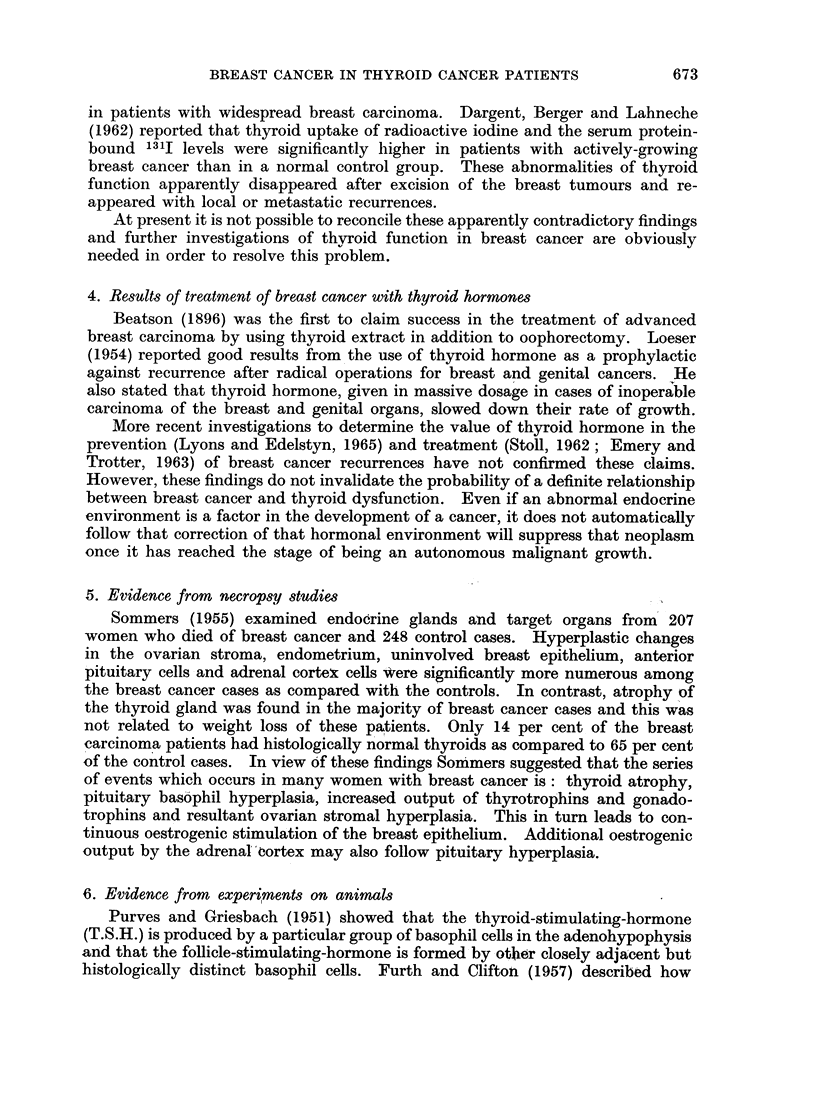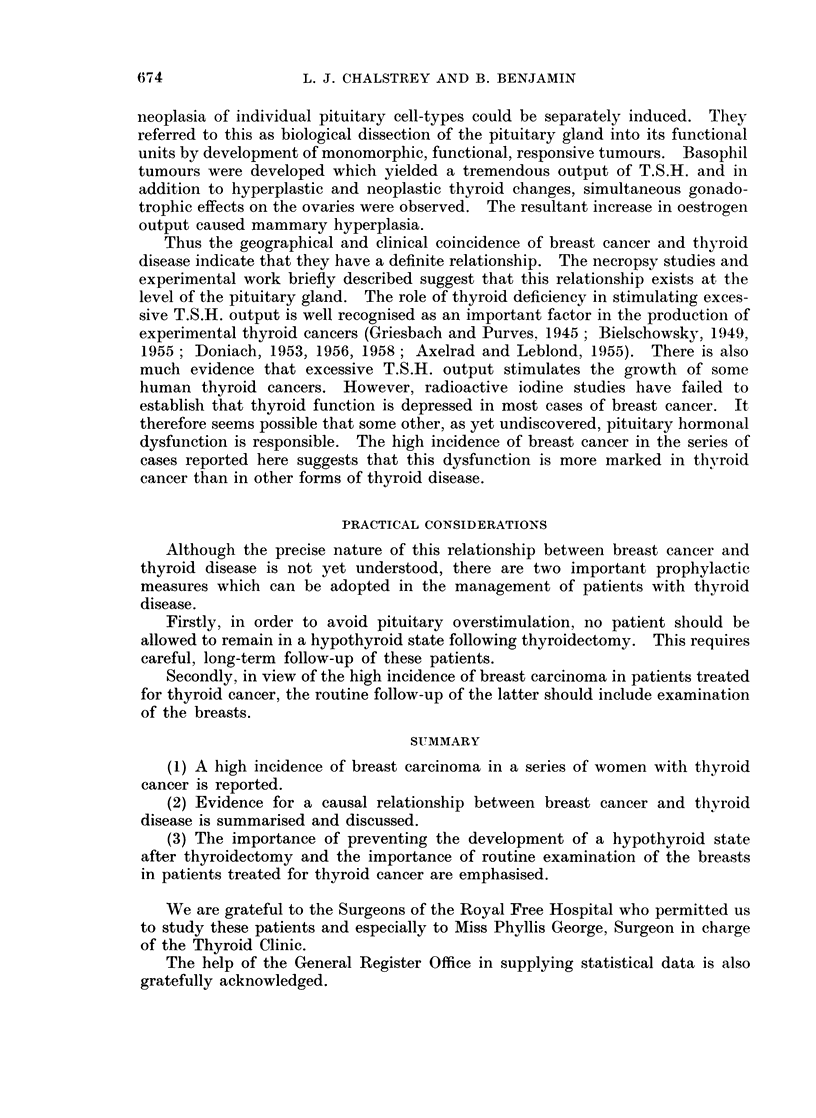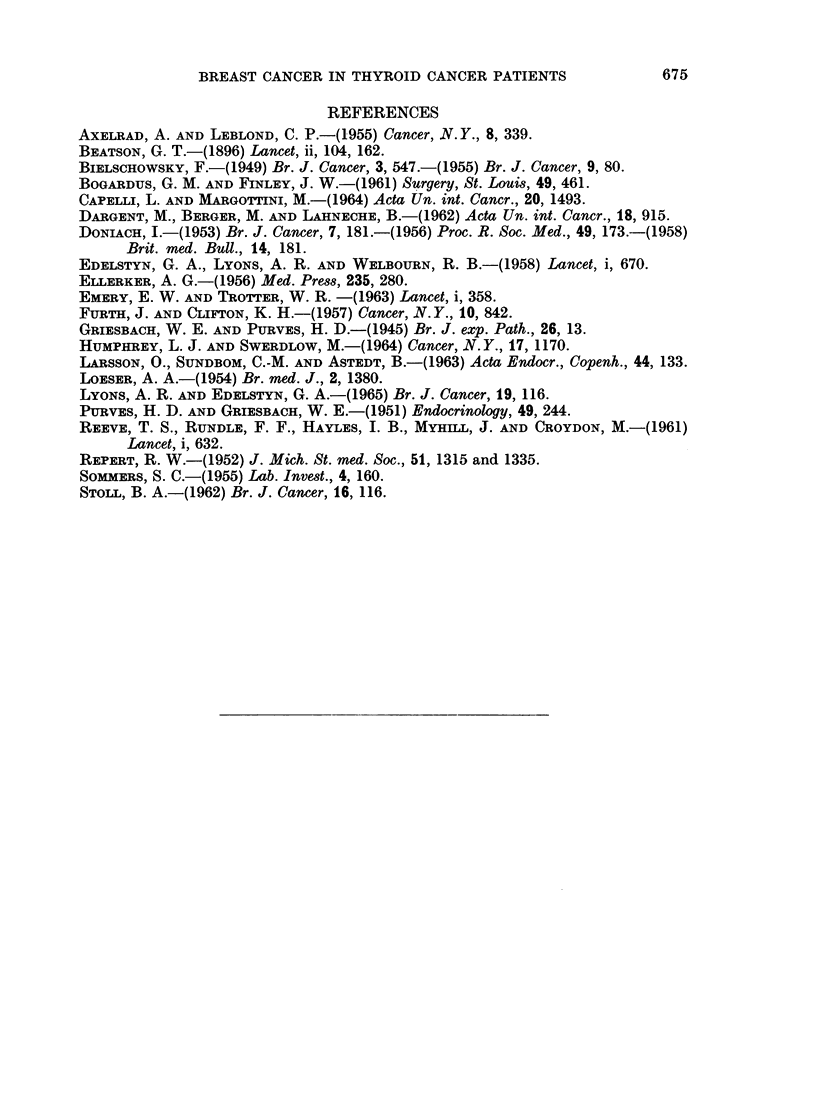# High incidence of breast cancer in thyroid cancer patients.

**DOI:** 10.1038/bjc.1966.78

**Published:** 1966-12

**Authors:** L. J. Chalstrey, B. Benjamin


### HIGH INCIDENCE OF BREAST CANCER IN THYROID CANCER PATIENTS

L. J. CHALSTREY AND B. BENJAMIN*

*From the Royal Free Hospital, Gray's Inn Road, London, W.C.1, and the Statistical Division, Ministry of Health, Russell Square, London, W.C.1*

Received for publication July 15, 1966

* Present address: Greater London Council, County Hall, Westminster, London, S.E.1.

A HIGH incidence of breast cancer has been found in a series of 92 female thyroid cancer patients. Careful examination of the literature has failed to reveal reports of similar findings in other thyroid cancer series.

During the twenty-year period 1945–1964, 106 patients with histologically proven thyroid cancer attended the Royal Free Hospital, London. Ninety-two of these were women and eight of these female patients also had primary carcinoma of the breast (8·7 per cent). Relevant details of their case histories are shown in Table I. In three of these patients the breast neoplasm preceded the thyroid cancer; in three cases the opposite occurred and in the other two, both lesions were found at the same time.

One patient (Case No. 5) was thyrotoxic before treatment. She received eight months Carbimazole therapy and then had a subtotal thyroidectomy. Histolo-

TABLE I.—*Thyroid and Breast Cancer*

| | Breast | | | Thyroid | | | | |
|---|---|---|---|---|---|---|---|---|
| Case No. | Age at diagnosis | Date of operation | Histology | Age at diagnosis | Date of operation | Histology | B.M.R. and Dates | $^{131}$I uptake and dates |
| 1 | 65 | 1963 | Carcinoma simplex | 53 | 1951 | Papillary carcinoma | 1952: −7%<br>1962: +1% | — |
| 2 | 49 | 1950 | Spheroidal cell carcinoma | 49 | 1950 | Papillary carcinoma | 1950: −8% | — |
| 3 | 49 | 1957 | Adeno-carcinoma | 47 | 1955 | Papillary carcinoma | 1956: ±0% | 1955 normal |
| 4 | 55 | 1938 | ? | 77 | 1960 | Papillary carcinoma | 1960: +1% | 1960 normal |
| 5 | 42 | 1965 | Mucus-secreting carcinoma | 37 | 1961 | Papillary carcinoma | 1961: +29%<br>1961: −14%<br>1963: +30%<br>1965: −30% | 1961 high |
| 6 | 32 | 1946 | Intraduct adeno-carcinoma | 48 | 1962 | Papillary carcinoma | 1962: −1% | 1962 low |
| 7 | 52 | 1941 | Spheroidal cell carcinoma | 63 | 1952 | Anaplastic carcinoma | 1952: −2% | 1952 normal |
| 8 | 76 | 1947 | Anaplastic scirrhous carcinoma | 76 | 1947 | Spindle cell sarcoma | — | — |

gical examination revealed a nodule of papillary adenocarcinoma in a hyperplastic gland. Following the operation her basal metabolic rate fell to $-14$ per cent and she was given 0·2 mg. l-thyroxine daily for the next two years. Mild symptoms suggestive of hyperthyroidism recurred and her basal metabolic rate was found to be $+30$ per cent. In view of this the l-thyroxine was discontinued. Two years later she noticed a lump in the right breast. This was a mucus-secreting carcinoma with metastases in the right axillary lymph nodes and was treated by radical mastectomy, Cyclophosphamide therapy and radiotherapy. Her basal metabolic rate at the time of discovery of the breast neoplasm was $-30$ per cent.

The other seven patients were clinically euthyroid and basal metabolic rate estimations were all in the lower part of the normal range. Radioactive iodine uptake studies were normal in three patients, (Cases No. 3, 4 and 7), low in one patient (No. 6) and were not done in the other three (No. 1, 2 and 8).

In six of these patients the thyroid carcinoma was of the papillary type.

### DISCUSSION

An 8·7 per cent incidence of breast carcinoma in this series of thyroid cancer patients contrasts with a very much lower incidence in comparable age groups of the general population of women in England and Wales, as is shown by the figures from the National Cancer Register in Table II.

TABLE II.—*Registrations of Carcinoma of the Breast—England and Wales* 1962

| Age group | Number of registrations | Rate per 100,000 women in age group |
|---|---|---|
| 0–14 | 1 | |
| 15–24 | 12 | 0·4 |
| 24–34 | 314 | 10·8 |
| 35–44 | 1757 | 55·2 |
| 45–54 | 3339 | 103·1 |
| 55–64 | 3554 | 120·7 |
| 65–74 | 2981 | 139·6 |
| 75 and over | 2202 | 174·7 |
| not stated | 16 | — |
| 35 and over | 13833 | 107·8 |

Expressed as a per cent rate (to compare with the 8·7 per cent quoted above) the incidence in 1962 was not higher than 0·2 per cent in any age group and over the whole range from age 35 was only 0·1 per cent. In this comparison 1962 registrations have been used because coverage of the registration system was less complete in earlier years. The year 1962 is not considered to be incomparable regarding breast cancer incidence, with the period of twenty years to which the thyroid series relates. So far as mortality reflects incidence, there has been no significant change in the two recent decades. The death rate per million living for females which was 389 in 1962, was 363 in 1952 and 336 in 1942 but when account is taken of the ageing of this population in the period, this apparent rise is reduced to insignificance. Certainly 0·1 per cent is not likely to be an understatement of the incidence of breast cancer in the general female population of age 35 and over. Even allowing for the fact that the 8·7 per cent incidence of breast cancer in the series is subject to a 95 per cent confidence interval ranging from 2·8 to 14·6 per cent, it is clear that there is a significant excess incidence of breast cancer in this series.

Although we have encountered no other reports of a high incidence of breast carcinoma in patients with thyroid cancer, the literature contains many references to the relationship between breast cancer and thyroid disease in general.

Evidence on the subject of a causal relationship between thyroid dysfunction and breast cancer may be summarised as follows:

1. *Geographical coincidence*

Statistics have shown that areas in which goitres are common tend to have a breast cancer mortality rate which is high. The reverse is true in areas where thyroid disease is uncommon (Bogardus and Finley, 1961).

2. *Clinical coincidence*

Bogardus and Finley (1961) reported that of 79 patients with breast cancer 42 also had some abnormality of the thyroid gland. Goitres were present in 37 cases and the others had either had a thyroid operation or thyroid therapy in the past. Most of their patients were euthyroid, none was hyperthyroid. Ellerker (1956) reported a 7·6 per cent incidence of goitres in 157 cases of breast cancer. In another group of 100 women between 40 and 60 years old, who had previously undergone thyroidectomy, he found a 6 per cent incidence of breast carcinoma. Only one of these thyroid operations had been for cancer, 30 were for thyrotoxicosis and the remaining 69 for various types of non-toxic goitre. On the other hand, Humphrey and Swerdlow (1964) found no incidence of breast carcinoma in a 12-year follow-up of 196 patients who had previously had thyroidectomy. Their finding of previous thyroid disease in 12 per cent of patients with breast cancer agrees with that of Repert (1952) who states that this is 10 times the expected rate for the incidence of thyroid disease.

Larsson, Sundbom and Astedt (1963) reported co-existent gynaecomastia in two patients with thyrotoxicosis and one with an active "malignant thyroid adenoma ". During treatment of the thyrotoxicosis the gynaecomastia disappeared completely in one case. Mastectomy was performed on the other two patients. They found an increased urinary output of 17-ketogenic steroids, gonadotrophins and oestrogens and suggested that these may have been due to the action of the thyrotrophic hormone of the pituitary gland on the adrenal cortex. There was no evidence of liver dysfunction in these patients.

3. *Evidence from investigations of thyroid function*

Using radioactive iodine ($^{131}I$) Edelstyn, Lyons and Welbourn (1958) studied thyroid function in patients with breast cancer and concluded that, whereas it was normal in patients with localised breast carcinoma, thyroid function was significantly lowered in those with widespread metastases. They suggested that this could be due either to the metastases depressing thyroid activity or to the reduced thyroid function favouring the distant spread of the cancer.

Later investigations of this sort (Reeve *et al.*, 1961 ; Capelli and Margottini, 1964) failed to confirm any statistically significant alteration of thyroid function in patients with widespread breast carcinoma. Dargent, Berger and Lahneche (1962) reported that thyroid uptake of radioactive iodine and the serum protein-bound $^{131}I$ levels were significantly higher in patients with actively-growing breast cancer than in a normal control group. These abnormalities of thyroid function apparently disappeared after excision of the breast tumours and re-appeared with local or metastatic recurrences.

At present it is not possible to reconcile these apparently contradictory findings and further investigations of thyroid function in breast cancer are obviously needed in order to resolve this problem.

4. *Results of treatment of breast cancer with thyroid hormones*

Beatson (1896) was the first to claim success in the treatment of advanced breast carcinoma by using thyroid extract in addition to oophorectomy. Loeser (1954) reported good results from the use of thyroid hormone as a prophylactic against recurrence after radical operations for breast and genital cancers. He also stated that thyroid hormone, given in massive dosage in cases of inoperable carcinoma of the breast and genital organs, slowed down their rate of growth.

More recent investigations to determine the value of thyroid hormone in the prevention (Lyons and Edelstyn, 1965) and treatment (Stoll, 1962 ; Emery and Trotter, 1963) of breast cancer recurrences have not confirmed these claims. However, these findings do not invalidate the probability of a definite relationship between breast cancer and thyroid dysfunction. Even if an abnormal endocrine environment is a factor in the development of a cancer, it does not automatically follow that correction of that hormonal environment will suppress that neoplasm once it has reached the stage of being an autonomous malignant growth.

5. *Evidence from necropsy studies*

Sommers (1955) examined endocrine glands and target organs from 207 women who died of breast cancer and 248 control cases. Hyperplastic changes in the ovarian stroma, endometrium, uninvolved breast epithelium, anterior pituitary cells and adrenal cortex cells were significantly more numerous among the breast cancer cases as compared with the controls. In contrast, atrophy of the thyroid gland was found in the majority of breast cancer cases and this was not related to weight loss of these patients. Only 14 per cent of the breast carcinoma patients had histologically normal thyroids as compared to 65 per cent of the control cases. In view of these findings Sommers suggested that the series of events which occurs in many women with breast cancer is : thyroid atrophy, pituitary basophil hyperplasia, increased output of thyrotrophins and gonado-trophins and resultant ovarian stromal hyperplasia. This in turn leads to continuous oestrogenic stimulation of the breast epithelium. Additional oestrogenic output by the adrenal cortex may also follow pituitary hyperplasia.

6. *Evidence from experiments on animals*

Purves and Griesbach (1951) showed that the thyroid-stimulating-hormone (T.S.H.) is produced by a particular group of basophil cells in the adenohypophysis and that the follicle-stimulating-hormone is formed by other closely adjacent but histologically distinct basophil cells. Furth and Clifton (1957) described how neoplasia of individual pituitary cell-types could be separately induced. They referred to this as biological dissection of the pituitary gland into its functional units by development of monomorphic, functional, responsive tumours. Basophil tumours were developed which yielded a tremendous output of T.S.H. and in addition to hyperplastic and neoplastic thyroid changes, simultaneous gonadotrophic effects on the ovaries were observed. The resultant increase in oestrogen output caused mammary hyperplasia.

Thus the geographical and clinical coincidence of breast cancer and thyroid disease indicate that they have a definite relationship. The necropsy studies and experimental work briefly described suggest that this relationship exists at the level of the pituitary gland. The role of thyroid deficiency in stimulating excessive T.S.H. output is well recognised as an important factor in the production of experimental thyroid cancers (Griesbach and Purves, 1945 ; Bielschowsky, 1949, 1955 ; Doniach, 1953, 1956, 1958 ; Axelrad and Leblond, 1955). There is also much evidence that excessive T.S.H. output stimulates the growth of some human thyroid cancers. However, radioactive iodine studies have failed to establish that thyroid function is depressed in most cases of breast cancer. It therefore seems possible that some other, as yet undiscovered, pituitary hormonal dysfunction is responsible. The high incidence of breast cancer in the series of cases reported here suggests that this dysfunction is more marked in thyroid cancer than in other forms of thyroid disease.

### PRACTICAL CONSIDERATIONS

Although the precise nature of this relationship between breast cancer and thyroid disease is not yet understood, there are two important prophylactic measures which can be adopted in the management of patients with thyroid disease.

Firstly, in order to avoid pituitary overstimulation, no patient should be allowed to remain in a hypothyroid state following thyroidectomy. This requires careful, long-term follow-up of these patients.

Secondly, in view of the high incidence of breast carcinoma in patients treated for thyroid cancer, the routine follow-up of the latter should include examination of the breasts.

### SUMMARY

(1) A high incidence of breast carcinoma in a series of women with thyroid cancer is reported.

(2) Evidence for a causal relationship between breast cancer and thyroid disease is summarised and discussed.

(3) The importance of preventing the development of a hypothyroid state after thyroidectomy and the importance of routine examination of the breasts in patients treated for thyroid cancer are emphasised.

We are grateful to the Surgeons of the Royal Free Hospital who permitted us to study these patients and especially to Miss Phyllis George, Surgeon in charge of the Thyroid Clinic.

The help of the General Register Office in supplying statistical data is also gratefully acknowledged.

### REFERENCES

Axelrad, A. and Leblond, C. P.—(1955) *Cancer, N.Y.*, **8**, 339.
Beatson, G. T.—(1896) *Lancet*, ii, 104, 162.
Bielschowsky, F.—(1949) *Br. J. Cancer*, **3**, 547.—(1955) *Br. J. Cancer*, **9**, 80.
Bogardus, G. M. and Finley, J. W.—(1961) *Surgery, St. Louis*, **49**, 461.
Capelli, L. and Margottini, M.—(1964) *Acta Un. int. Cancr.*, **20**, 1493.
Dargent, M., Berger, M. and Lahneche, B.—(1962) *Acta Un. int. Cancr.*, **18**, 915.
Doniach, I.—(1953) *Br. J. Cancer*, **7**, 181.—(1956) *Proc. R. Soc. Med.*, **49**, 173.—(1958) *Brit. med. Bull.*, **14**, 181.
Edelstyn, G. A., Lyons, A. R. and Welbourn, R. B.—(1958) *Lancet*, i, 670.
Ellerker, A. G.—(1956) *Med. Press*, **235**, 280.
Emery, E. W. and Trotter, W. R. —(1963) *Lancet*, i, 358.
Furth, J. and Clifton, K. H.—(1957) *Cancer, N.Y.*, **10**, 842.
Griesbach, W. E. and Purves, H. D.—(1945) *Br. J. exp. Path.*, **26**, 13.
Humphrey, L. J. and Swerdlow, M.—(1964) *Cancer, N.Y.*, **17**, 1170.
Larsson, O., Sundbom, C.-M. and Astedt, B.—(1963) *Acta Endocr., Copenh.*, **44**, 133.
Loeser, A. A.—(1954) *Br. med. J.*, **2**, 1380.
Lyons, A. R. and Edelstyn, G. A.—(1965) *Br. J. Cancer*, **19**, 116.
Purves, H. D. and Griesbach, W. E.—(1951) *Endocrinology*, **49**, 244.
Reeve, T. S., Rundle, F. F., Hayles, I. B., Myhill, J. and Croydon, M.—(1961) *Lancet*, i, 632.
Repert, R. W.—(1952) *J. Mich. St. med. Soc.*, **51**, 1315 and 1335.
Sommers, S. C.—(1955) *Lab. Invest.*, **4**, 160.
Stoll, B. A.—(1962) *Br. J. Cancer*, **16**, 116.